# Social Stigma and Knowledge of Tuberculosis and HIV among Patients with Both Diseases in Thailand

**DOI:** 10.1371/journal.pone.0006360

**Published:** 2009-07-23

**Authors:** Sirinapha X. Jittimanee, Sriprapa Nateniyom, Wanitchaya Kittikraisak, Channawong Burapat, Somsak Akksilp, Nopphanath Chumpathat, Chawin Sirinak, Wanchai Sattayawuthipong, Jay K. Varma

**Affiliations:** 1 Thailand Ministry of Public Health, Nonthaburi, Thailand; 2 Thailand Ministry of Public Health - U.S. Centers for Disease Control and Prevention Collaboration, Nonthaburi, Thailand; 3 Office of Disease Prevention and Control 7, Ubon Ratchathani, Thailand; 4 Bamrasnaradura Infectious Diseases Institute, Nonthaburi, Thailand; 5 Department of Health, Bangkok Metropolitan Administration, Bangkok, Thailand; 6 Phuket Provincial Health Office, Phuket, Thailand; 7 U.S. Centers for Disease Control and Prevention, Atlanta, Georgia, United States of America; McGill University Health Center, Montreal Chest Institute, Canada

## Abstract

**Introduction:**

Disease-related stigma and knowledge are believed to be associated with patients' willingness to seek treatment and adherence to treatment. HIV-associated tuberculosis (TB) presents unique challenges, because TB and HIV are both medically complex and stigmatizing diseases. In Thailand, we assessed knowledge and beliefs about these diseases among HIV-infected TB patients.

**Methods:**

We prospectively interviewed and examined HIV-infected TB patients from three provinces and one national referral hospital in Thailand from 2005–2006. At the beginning of TB treatment, we asked patients standardized questions about TB stigma, TB knowledge, and HIV knowledge. Responses were grouped into scores; scores equal to or greater than the median score of study population were considered high. Multiple logistic regression analysis was used to identify factors associated with scores.

**Results:**

Of 769 patients enrolled, 500 (65%) reported high TB stigma, 177 (23%) low TB knowledge, and 379 (49%) low HIV knowledge. Patients reporting high TB stigma were more likely to have taken antibiotics before TB treatment, to have first visited a traditional healer or private provider, to not know that monogamy can reduce the risk of acquiring HIV infection, and to have been hospitalized at enrollment. Patients with low TB knowledge were more likely to have severe TB disease, to be hospitalized at enrollment, to be treated at the national infectious diseases referral hospital, and to have low HIV knowledge. Patients with low HIV knowledge were more likely to know a TB patient and to have low TB knowledge.

**Discussion:**

We found that stigma and low disease-specific knowledge were common among HIV-infected TB patients and associated with similar factors. Further research is needed to determine whether reducing stigma and increasing TB and HIV knowledge among the general community and patients reduces diagnostic delay and improves patient outcomes.

## Introduction

For almost 30 years, the epidemics of tuberculosis (TB) and HIV have acted synergistically to produce excess illness and death around the world.[1] The TB/HIV “syndemic” presents a major challenge to public health programs, because rising rates of HIV lead to rising rates of TB disease, routine diagnostic methods often fail to diagnose TB in HIV-infected patients, and the case-fatality rate for HIV-associated TB is high.[1], [2] In addition to the biomedical challenges, the TB/HIV syndemic also challenges public health programs to deal with important social and cultural factors linked to both diseases. Both diseases are deeply linked to poverty and lack of access to high-quality medical care.[3] Groups that are at high risk of acquiring HIV infection—including injection drug use, prisoners, mobile populations—are also at high risk of TB disease, and ensuring adherence to TB or HIV treatment is particularly challenging in these groups.[4], [5], [6], [7], [8], [9] In their advanced form, both TB and HIV lead to severe weight loss, fever, and night sweats, frequently leading TB patients to think they have HIV and vice versa.[10]

Because of the close social interplay between TB and HIV, public health professionals are increasingly interested in factors, such as disease-related stigma and knowledge, that might impact prevention and treatment of both diseases.[11] Although TB and HIV have many biomedical differences, the origins and impact of stigma are similar. Factors that drive stigma for both diseases are that TB and HIV produce severe illness, can be transmitted to others, and occur more frequently in populations perceived to be different or deviant.[12], [13] Stigma of either disease can lead to isolation from friends and family, loss of employment, exclusion from community activities, and fear of seeking out medical care.[14], [15], [16], [17], [18] Knowledge about these diseases is believed to be an important determinant of health-seeking behavior as well as adherence to preventive measures and treatment.[19], [20] Nevertheless, knowledge of both diseases may be limited, even in patients with both diseases.[21], [22]

Thailand has a severe TB/HIV syndemic. An estimated 600,000 people are living with HIV/AIDS, 90,000 TB patients are diagnosed annually, 15–20% of all TB cases are HIV positive, and the case-fatality rate of HIV-associated TB is high.[23], [24], [25], [26] Two studies conducted in selected facilities from two Thai provinces have attempted to assess TB and HIV stigma[13], [27], but none have evaluated a large cohort of HIV-infected TB patients derived from multiple facilities and provinces. In this study, we assessed TB stigma, TB knowledge, and HIV knowledge among HIV-infected TB patients in Thailand.

## Methods

### Study population

We enrolled HIV-infected TB patients from 32 public TB treatment facilities in Bangkok, Phuket, and Ubon Ratchathani provinces and from the national infectious diseases referral hospital (Bamrasnaradura Infectious Diseases Institute) in Nonthaburi province. The study population included adults aged ≥18 years with documented HIV infection who were diagnosed with active TB disease according to national TB program guidelines, registered for TB treatment at one of the participating facilities, and received anti-TB therapy for <4 weeks before study enrollment. All TB patients, including persons that had relapsed and those that returned after default, were included. Prisoners were excluded, because their follow-up through the duration of the study could not be guaranteed. Pregnant women were excluded, because their care involved multiple providers. At the beginning of TB treatment, patients who consented to study enrollment underwent a physical examination and answered questions about demographic characteristics, past and present medical history, knowledge and attitudes related to TB and HIV, and sex and drug use history. For this study, patients received usual care for TB, HIV, and other diseases, and no health-related interventions were performed. All participants gave written consent and this study was approved by the ethical review committees of the Bangkok Metropolitan Administration, the Thailand Ministry of Public Health, and the U.S. Centers for Disease Control and Prevention.

### Measurement of stigma and knowledge

Table 1 shows the questions used to assess stigma and knowledge. We measured TB stigma using four questions. An answer consistent with TB stigma was scored with one point. An answer not consistent with stigma or a missing response was scored as zero points. A total stigma score was created by summing the scores for all questions. The score ranged from 0 to 4, with the higher the score, the greater the degree of TB stigma.

**Table 1:** Baseline TB stigma, TB knowledge, and HIV knowledge among HIV-infected TB patients in Thailand.

Stigma and knowledge questions	n^a^	Number of patients with answer consistent with stigma	Number of patients with incorrect answer	(%)
*TB stigma* b				
If someone in your family had TB, you would share a plate with them, i.e., eat together	738	292		(40)
If someone in your family had received treatment for TB, you would share the same bed with them	742	254		(34)
Having TB would be an embarrassment in your family	757	174		(23)
Having TB would be an embarrassment in your community	734	247		(34)
*TB knowledge* c				
A person with TB can be cured	739		30	(4)
Someone who has TB always has HIV/AIDS	690		111	(16)
You can die from TB if you do not take your drugs regularly	738		69	(9)
TB treatment usually takes at least one year	719		415	(58)
You should keep taking your medicine if you develop severe nausea, severe vomiting, or a skin rash during TB treatment	735		319	(43)
You should stop taking TB drugs as soon as you feel better	753		103	(14)
You can continue your TB treatment even if you transfer to another hospital closer to your home	748		48	(6)
*HIV knowledge* d				
The risk of getting HIV/AIDS can be reduced by having sex with only one faithful uninfected partner	751		63	(8)
The risk of getting HIV/AIDS can be reduced by using condoms	760		10	(1)
A healthy-looking person can have HIV/AIDS	741		114	(15)
A person can get HIV from mosquito bites	729		92	(13)
A person can get HIV by sharing a meal with someone who has HIV	746		152	(20)

TB, tuberculosis; HIV, human immunodeficiency virus; AIDS, acquired immunodeficiency syndrome.

a Those with available answers.

b Five hundred patients had high TB stigma; 75 did not respond to one or more TB stigma questions.

c One hundred and seventy-seven had low TB knowledge; 171 did not respond to one or more TB knowledge questions.

d Three hundred and seventy-nine patients had low HIV knowledge; 69 did not respond to one or more HIV knowledge questions.

We measured TB knowledge using seven questions. These questions were derived from information routinely provided to patients as part of the national TB program. A correct response was scored with one point. Incorrect or missing responses were scored with zero points. A total TB knowledge score was created by summing the scores for all questions. The score ranged from 0 to 7, with the higher the score, the greater the patient's TB knowledge.

We measured HIV knowledge using five questions. These questions were recommended by the Joint United Nation's Program on HIV/AIDS (UNAIDS) for monitoring a community's knowledge about HIV transmission and preventive health behaviors.[28] A correct response was scored with one point. Incorrect or missing responses were scored with zero points. A total HIV knowledge score was created by summing the scores for all questions. The score ranged from 0 to 5, with the higher the score, the greater the patient's HIV knowledge.

### Other definitions

We classified patients as having “severe TB” if they had extra-pulmonary TB other than peripheral lymphatic or pleural TB or if they had all of the following characteristics: self-reported weight loss >10% of body weight, coughing up blood, difficulty breathing in past four weeks before TB diagnosis, and either cavitary TB or >1/3 involvement of either lung on the initial chest radiograph. We classified patients as having delayed TB diagnosis if they reported having a cough lasting greater than one month before TB diagnosis or having other symptoms that lasted longer than 14 days and self-assessed these symptoms as being severe.

### Statistical analysis

Stigma or knowledge scores equal to or greater than the median score of study population were considered high. To identify factors associated with high stigma, low TB knowledge, and low HIV knowledge, we first identified factors associated with each outcome variable at p≤0.20 in bivariable analysis, tested them for co-linearity, and then constructed multiple logistic regression models. Two-way interaction terms were generated as products of covariates and entered in the models. For each multiple logistic regression analysis, we fitted two models: a parsimonious model using backward stepwise variable selection and a saturated model. We assessed model fitness using the Hosmer-Lemeshow goodness-of-fit test. Findings from both models were similar, and we chose to report estimates from the saturated model. No interaction terms were included in the final models. We defined a two-sided p-value of ≤0.05 as statistical significance. All analyses were performed by Stata software version 8.0 (StataCorp LP, College Station, TX , U.S.A.).

## Results

### Enrollment and baseline characteristics of patients

From May 2005 to September 2006, 1,096 HIV-infected TB patients were eligible for the study; of these, 849 (77%) enrolled. Reasons for not enrolling were refusal (1245; 50%), death before enrollment (21; 8%), visiting during non-operation hours or after completion of enrollment (84; 34%), self-reported to be too ill (14; 6%), and communication problems (4; 2%). After excluding 80 patients who subsequently were diagnosed as not having TB, we analyzed data for 769 patients.

The median scores for TB stigma, TB knowledge, and HIV knowledge were 1 (interquartile [IQR], 0–2), 5 (IQR, 5–6), and 5 (IQR, 4–5), respectively. **Table 2** summarizes characteristics of patients enrolled in the study and characteristics stratified by patients with high TB stigma, low TB knowledge, and low HIV knowledge. The median age was 34 years (IQR, 30–41), and 538 (70%) were male. Almost 70% were diagnosed with pulmonary TB. Both delayed TB diagnosis (48%) and severe TB disease (42%) were common. Almost half knew that they were HIV-infected before they were diagnosed with TB, and over 80% had a CD4+ T-lymphocyte count <200 cells/µL.

**Table 2:** Patient characteristics stratified by TB stigma, TB knowledge, and HIV knowledge.

Characteristics	All (n = 769)		High TB stigma^d^ (n = 500)		Low TB knowledge^d^ (n = 177)		Low HIV knowledge^d^ (n = 379)	
	n	(%)	n	(%)	n	(%)	n	(%)
Male	538	(70)	351	(70)	127	(71)	266	(70)
Age>34 years old	380	(49)	249	(50)	88	(50)	185	(49)
TB disease classification								
Pulmonary TB	537	(70)	365	(73)	108	(61)	253	(67)
Extra-pulmonary TB	232	(30)	135	(27)	69	(39)	126	(33)
CD4<200 cells/µL^a^	609	(81)	395	(82)	154	(88)	302	(82)
Site								
Bangkok	177	(23)	121	(24)	20	(11)	86	(23)
National hospital	228	(30)	153	(31)	89	(50)	103	(27)
Ubon Ratchathani	254	(33)	164	(33)	42	(24)	135	(36)
Phuket	110	(14)	62	(12)	26	(15)	55	(14)
Severe TB disease^b^	321	(42)	199	(40)	103	(58)	169	(45)
Hospitalized at enrollment	205	(27)	142	(28)	75	(42)	96	(25)
Delay in TB diagnosis^c^	368	(48)	243	(49)	76	(43)	178	(47)
Visited traditional healer or private hospital first	181	(23)	136	(27)	48	(27)	99	(26)
Diagnosed with HIV before TB diagnosis	363	(47)	227	(45)	91	(51)	186	(49)
Know anyone with TB	282	(37)	181	(36)	55	(31)	154	(41)
Low TB knowledge	177	(23)	116	(23)	-	-	115	(30)
High TB stigma	500	(65)	-	-	116	(65)	253	(67)
Low HIV knowledge	379	(49)	253	(51)	115	(65)	-	-
Household has safe drinking water source	558	(73)	355	(71)	135	(76)	278	(73)
Household has a motorcycle	467	(61)	289	(58)	110	(62)	228	(60)
Household has a car	183	(24)	110	(22)	44	(25)	87	(23)
Household has a telephone	564	(73)	372	(74)	129	(73)	264	(70)
Had sexual intercourse in the past 6 months	335	(44)	217	(43)	75	(42)	156	(41)
Ever drank alcohol	538	(70)	346	(69)	129	(73)	247	(65)
Drank alcohol in past 3 months	181	(23)	110	(22)	39	(22)	76	(20)

TB, tuberculosis; HIV, human immunodeficiency virus; CD4, CD4+ T-lymphocyte.

a Those with available results only.

b Patients who had extra-pulmonary TB other than peripheral lymphatic TB or had all of the following characteristics: self-reported weight loss >10% of body weight, coughing up blood, difficulty breathing in past 4 weeks before TB diagnosis, and cavitary TB or >1/3 involvement of either lung at the initial evaluation.

c Patients who reported having a cough lasting greater than one month before TB diagnosis or had other symptoms that lasted longer than 14 days and self-assessed these symptoms as being severe.

d High TB stigma defined as TB stigma score ≥1; low TB knowledge defined as TB knowledge score<5; and low HIV knowledge defined as HIV knowledge score<5.

### TB stigma

High TB stigma was found in 500 (65%) patients. About 34% reported that having TB would be an embarrassment in the community, and 23% agreed that having TB would be an embarrassment in the family.**[** **Table 1** **]** In multiple logistic regression analysis, patients reporting high TB stigma were more likely to have taken antibiotics before TB treatment (adjusted odds ratio [aOR], 1.5; 95% confidence interval [CI], 1.0–2.1), to have first visited a private provider (aOR, 1.7; CI, 1.1–2.6), to not know that monogamy can reduce the risk of acquiring HIV infection (aOR, 2.2; CI, 1.1–4.2), and to have been hospitalized at enrollment (aOR, 1.7; CI, 1.1–2.6).**[** **Table 3** **]**

**Table 3:** Bivariable and multiple logistic regression analyses of predictors for having high TB stigma^a^ among HIV-infected TB patients.

Characteristics	OR	95% CI	p	AOR	95% CI	p
Age	1.0	1.0–1.0	0.25	1.0	1.0–1.0	0.43
Male	1.0	0.7–1.4	0.81	1.1	0.8–1.6	0.57
Treated at national infectious diseases referral hospital^b^	0.9	0.6–1.4	0.81	1.1	0.6–1.8	0.81
Treated at Ubon Ratchathani^b^	0.8	0.6–1.3	0.43	0.9	0.5–1.6	0.81
Treated at Phuket^b^	0.6	0.3–0.9	0.03	0.6	0.3–1.1	0.10
Severe TB disease^c^	0.8	0.6–1.1	0.14	0.8	0.6–1.1	0.22
Hospitalized at enrollment	1.3	0.9–1.8	0.14	1.7	1.1–2.6	0.01
Drank alcohol in past 3 months	0.8	0.5–1.1	0.16	0.8	0.5–1.1	0.22
Ever used sleeping pill	1.4	0.9–2.1	0.09	1.3	0.8–2.0	0.29
Took antibiotics in past 4 weeks	1.3	0.9–1.8	0.10	1.5	1.0–2.1	0.04
Visited traditional healer/private provider first	1.9	1.3–2.7	<0.01	1.7	1.1–2.6	0.01
Diagnosed with HIV before TB	0.8	0.6–1.1	0.17	0.9	0.7–1.3	0.76
Household has safe drinking water Source	0.8	0.6–1.1	0.19	0.7	0.4–1.0	0.06
Household has a motorcycle	0.7	0.5–1.0	0.03	0.8	0.5–1.1	0.14
Household has a car	0.8	0.5–1.1	0.12	0.8	0.5–1.1	0.18
Answer incorrectly on question the risk of getting HIV/AIDS can be reduced by having sex with only one faithful, uninfected partner	2.2	1.2–4.1	0.01	2.2	1.1–4.2	0.02
Answered incorrectly on question a person can get HIV by sharing a meal with someone who has HIV	1.7	1.0–2.8	0.03	1.5	0.9–2.5	0.15

TB, tuberculosis; HIV, human immunodeficiency virus; OR, odds ratio; AOR, adjusted odds ratio; CI, confidence interval; variables for which p≤0.20 in bivariable analyses and potential confounders were included in multiple logistic regression analysis.

a TB stigma score ≥1; TB stigma score is a summary score of the number of TB stigma questions (see Table 1; each question was worth 1 point) that a patient answered consistent with stigma.

b Compared with being treated in Bangkok.

c Patients who had extra-pulmonary TB other than peripheral lymphatic TB or had all of the following characteristics: self-reported weight loss >10% of body weight, coughing up blood, difficulty breathing in past 4 weeks before TB diagnosis, and cavitary TB or >1/3 involvement of either lung at the initial evaluation.

### TB knowledge

Low TB knowledge was found in 177 (23%) patients. The most common incorrect answers involved the appropriate action to take if they had a severe medication side effect and the duration of TB treatment in Thailand.**[** **Table 1** **]** In multiple logistic regression analysis, patients with low TB knowledge were more likely to have severe TB disease (aOR, 1.7; CI, 1.1–2.5), to be hospitalized at enrollment (aOR, 1.7; CI, 1.1–2.6), to be treated at the national infectious diseases referral hospital (aOR, 3.0; CI, 1.6–5.8), and to have low HIV knowledge (aOR, 2.2; CI, 1.5–3.3).**[** **Table 4** **]**

**Table 4:** Bivariable and multiple logistic regression analyses of predictors for having low TB knowledge^a^ among HIV-infected TB patients.

Characteristics	OR	95% CI	p	AOR	95% CI	p
Age	1.0	1.0–1.0	0.96	1.0	1.0–1.0	0.92
Male	1.1	0.8–1.6	0.57	1.2	0.8–1.9	0.33
Treated at national infectious diseases referral hospital^b^	5.1	3.0–8.7	<0.01	3.0	1.6–5.8	<0.01
Treated at Ubon Ratchathani^b^	1.5	0.9–2.7	0.13	0.8	0.4–1.5	0.44
Treated at Phuket^b^	2.4	1.3–4.0	0.01	1.6	0.8–3.4	0.20
Severe TB disease^§^	2.4	1.7–3.4	<0.01	1.7	1.1–2.5	0.02
CD4<200 cells/µL at baseline	2.1	1.3–3.5	<0.01	1.7	0.9–3.1	0.10
Hospitalized at enrollment	2.6	1.8–3.7	<0.01	1.7	1.1–2.6	0.02
Know anyone with TB	0.8	0.5–1.1	0.13	0.8	0.5–1.3	0.38
Low HIV knowledge	2.3	1.6–3.3	<0.01	2.2	1.5–3.3	<0.01
Delay in TB diagnosis^c^	0.8	0.5–1.1	0.14	0.8	0.6–1.3	0.41
Registered as new case	1.9	1.1–3.3	0.03	1.2	0.6–2.3	0.57
Answered negatively on question if someone in your family received treatment for TB, you would share the same bed with them	1.4	1.0–2.0	0.06	1.2	0.8–1.8	0.36

TB, tuberculosis; HIV, human immunodeficiency virus; OR, odds ratio; AOR, adjusted odds ratio; CI, confidence interval; CD4, CD4+ T-lymphocyte; variables for which p≤0.20 in bivariable analyses and potential confounders were included in multiple logistic regression analysis.

a TB knowledge score<5; TB knowledge score is a summary score of the number of TB knowledge questions (see table 2 - each question is worth 1 point) that a patient correctly answered.

b Compared with being treated in Bangkok.

c Patients who had extra-pulmonary TB other than peripheral lymphatic TB or had all of the following characteristics: self-reported weight loss >10% of body weight, coughing up blood, difficulty breathing in past 4 weeks before TB diagnosis, and cavitary TB or >1/3 involvement of either lung at the initial evaluation.

### HIV knowledge

Low HIV knowledge was found in 379 (49%). The question most frequently answered wrong was about the risk of HIV transmission from sharing a meal with an HIV-infected person; 152 patients (20%) believed that this was possible.**[** **Table 1** **]** Of 700 patients with no missing replies to all HIV knowledge questions, 390 (56%) correctly answered all five questions. In multiple logistic regression analysis, patients with low HIV knowledge were more likely to know a TB patient, (aOR, 1.5; CI, 1.1–2.1) and to have low TB knowledge (aOR, 2.3; CI, 1.5–3.4); they were less likely to ever drink alcohol (aOR, 0.59; CI, 0.41–0.86) or to have a household telephone (aOR, 0.69; CI, 0.48–0.98).**[** **Table 5** **]**

**Table 5:** Bivariable and multiple logistic regression analyses of predictors for having low HIV knowledge^a^ among HIV-infected TB patients.

Characteristics	OR	95% CI	p	AOR	95% CI	p
Age	1.0	1.0–1.0	0.84	1.0	1.0–1.0	0.96
Male	1.0	0.7–1.4	0.94	1.3	0.9–1.8	0.19
Treated at national infectious diseases referral hospital^b^	0.9	0.6–1.3	0.55	0.8	0.5–1.3	0.45
Treated at Ubon Ratchathani^b^	1.2	0.8–1.8	0.33	1.3	0.8–2.1	0.22
Treated at Phuket^b^	1.0	0.6–1.6	0.93	1.1	0.6–1.9	0.73
Know anyone with TB	1.4	1.1–1.9	0.02	1.5	1.1–2.0	0.02
Severe TB disease^c^	1.3	0.9–1.7	0.12	1.2	0.8–1.6	0.31
Low TB knowledge	2.3	1.6–3.3	<0.01	2.3	1.5–3.3	<0.01
Ever drank alcohol	0.6	0.5–0.9	0.01	0.6	0.4–0.9	0.01
Drank alcohol in past 3 months	0.7	0.5–1.0	0.03	0.7	0.5–1.1	0.14
Ever used sleeping pill	1.3	0.9–1.9	0.19	1.4	0.9–2.1	0.15
Took antibiotics in past 4 weeks	1.3	1.0–1.7	0.10	1.3	1.0–1.9	0.09
Visited traditional healer/private hospital first	1.3	1.0–1.9	0.09	1.3	0.9–1.9	0.18
Household has a telephone	0.7	0.5–1.0	0.03	0.7	0.5–1.0	0.04
Had sexual intercourse in past 6 months	0.8	0.6–1.1	0.19	0.9	0.6–1.2	0.51

TB, tuberculosis; HIV, human immunodeficiency virus; OR, odds ratio; AOR, adjusted odds ratio; CI, confidence interval; variables for which p≤0.20 in bivariable analyses and potential confounders were included in multiple logistic regression analysis.

a HIV knowledge score<5; HIV knowledge score is a summary score of the number of HIV knowledge questions (see table 2 - each question is worth 1 point) that a patient correctly answered.

b Compared with being treated in Bangkok.

c Patients who had extra-pulmonary TB other than peripheral lymphatic TB or had all of the following characteristics: self-reported weight loss >10% of body weight, coughing up blood, difficulty breathing in past 4 weeks before TB diagnosis, and cavitary TB or >1/3 involvement of either lung at the initial evaluation.

## Discussion

In this large, diverse cohort from Thailand, we found that HIV-infected TB patients frequently reported attitudes consistent with high TB stigma and had important knowledge gaps about TB and HIV.

In our study, high TB stigma was independently associated with patients taking antibiotics before they sought TB treatment and with first seeking care from a private practitioner. After being diagnosed with TB, patients with high TB stigma were also more likely to have been hospitalized for TB, a marker of disease severity. Two of the most important indicators of TB program performance—case finding and treatment success—may, therefore, be adversely impacted by stigma. Self-treatment for TB or receipt of care outside the public TB control system could reduce case finding, since cases from the private sector are often not reported, and severe TB disease could lead to worse TB treatment outcomes.[29] This problem is beginning to receive more attention. In its Second Global Plan to Stop TB, World Health Organization emphasizes that case finding and treatment outcomes can be improved by community education and outreach to reduce TB-related stigma and discrimination.[30] Multiple studies from the past 10 years have documented the importance of addressing community perceptions of TB, rather than simply individual patient or family members' attitudes.[13], [15], [16], [27], [31] At least one study in Nicaragua has shown that TB-related stigma leads patients to conceal their TB diagnosis from others, reducing adherence and treatment completion rates.[17] Unfortunately, evidence-based strategies for reducing either patient or community-wide stigma are lacking. Self-help, advocacy, and support groups have been recommended, but their impact at a population-level and on TB control has not been firmly established.[32], [33]

We found that disease-specific knowledge may need to be improved among HIV-infected TB patients in Thailand. We would have expected patients' knowledge about TB disease to have been high, because patients were enrolled in this study after being registered for TB treatment and the TB registration process in Thailand involves standardized patient education. We found that low TB knowledge was closely associated with patients who had severe illness, i.e., high TB severity score, hospitalized, treated at the national infectious diseases referral hospital. Possible explanations for this association include that patients received less TB-specific education or retained less information from TB-specific education, because they were so severely ill. It is also possible that patients with less TB knowledge may be less likely to seek care until they are severely ill, although we did not find a relationship between TB diagnostic delay and knowledge to support this hypothesis. We found that HIV-specific knowledge was high, likely due to Thailand's national initiatives to prevent HIV infection.[34] Nevertheless, a surprisingly large number of patients incorrectly thought that mosquito bites and sharing a meal could transmit HIV and that all HIV patients must look sick. As with TB diagnosis, patients in this study already knew their HIV diagnosis and had received post-test HIV counseling. Our finding of a relationship between low TB knowledge and low HIV knowledge suggests that TB clinic staff should consider expanding the depth and frequency of patient education about both diseases.

Our study is subject to important limitations. First, no gold standards exist for measuring TB stigma, TB knowledge, and HIV knowledge. Because this analysis was embedded within a larger study, we used a standardized approach, involving quantitative analysis of a small number of questions. One recent study in Thailand developed a standardized set of questions and scoring system for quantifying TB and HIV stigma among TB patients.[11] A more complete assessment of stigma and knowledge in our study population would require use of such standardized, quantitative instruments or of extensive patient interviews and qualitative analysis. Second, we do not have sufficient data to assess the true public health importance of the scores that we created. Although we found statistically significant associations that were epidemiologically plausible, we do not have independent data to validate the public health significance of our stigma and knowledge scores, e.g., do patients with a high stigma score truly have higher levels of stigmatizing beliefs?

In conclusion, we found that stigma and low disease-specific knowledge were common among HIV-infected TB patients and associated with similar factors, such as TB disease severity. Further research is needed to determine whether reducing stigma and increasing TB and HIV knowledge among the general community and patients reduces diagnostic delays and improves patient outcomes.
